# Comparison and agreement between two image analysis tools for quantifying GFP::SNB-1 puncta in *fshr-1* mutants of C. elegans

**DOI:** 10.17912/micropub.biology.001005

**Published:** 2023-12-14

**Authors:** Heino Hulsey-Vincent, Makenzi McClain, Morgan Buckley, Jennifer R. Kowalski, Caroline L. Dahlberg

**Affiliations:** 1 Biology, Western Washington University, Bellingham, Washington, United States; 2 Biological Sciences, Butler University, Indianapolis, Indiana, United States

## Abstract

Quantitative imaging of synaptic vesicle localization and abundance using fluorescently labeled synaptic vesicle associated proteins like GFP::SNB-1 is a well-established method for measuring changes in synapse structure at neuromuscular junctions (NMJ) in *C. elegans* . To date, however, the ability to easily and reproducibly measure key parameters at the NMJ – maximum intensity, size of GFP::SNB-1 puncta, density of puncta – has relied on the use of expensive, customizable software that requires coding skills to modify, precluding widespread access and thus preventing standardization within the field. We carried out a comparative evaluation of a new, open-source Fiji puncta plugin versus traditional Igor-based analysis of GFP::SNB-1 imaging data taken of cholinergic motor neurons in the dorsal nerve cord of loss of function mutants in *fshr-1* , which encodes a G protein-coupled receptor known to impact GFP::SNB-1 accumulation. We analyzed images taken on a widefield fluorescence microscope, as well as on a spinning disk confocal microscope. Our data demonstrate strong concordance between the differences in GFP::SNB-1 localization in *fshr-1* mutants compared to wild type worms across both analysis platforms (Fiji and Igor), as well as across microscope types (widefield and confocal). These data also agree with previously published observations related to synapse number and GFP::SNB-1 intensity in *fshr-1* and wild type worms. Based on these findings, we conclude that the Fiji platform is viable as a method for analyzing synaptic vesicle localization and abundance at cholinergic dorsal nerve cord motor NMJs and expect the Fiji puncta plugin to be of broad utility in imaging across a variety of imaging platforms and synaptic markers.

**Figure 1. Comparison of analyses of widefield and confocal images of GFP::SNB-1 using Igor and Fiji.**
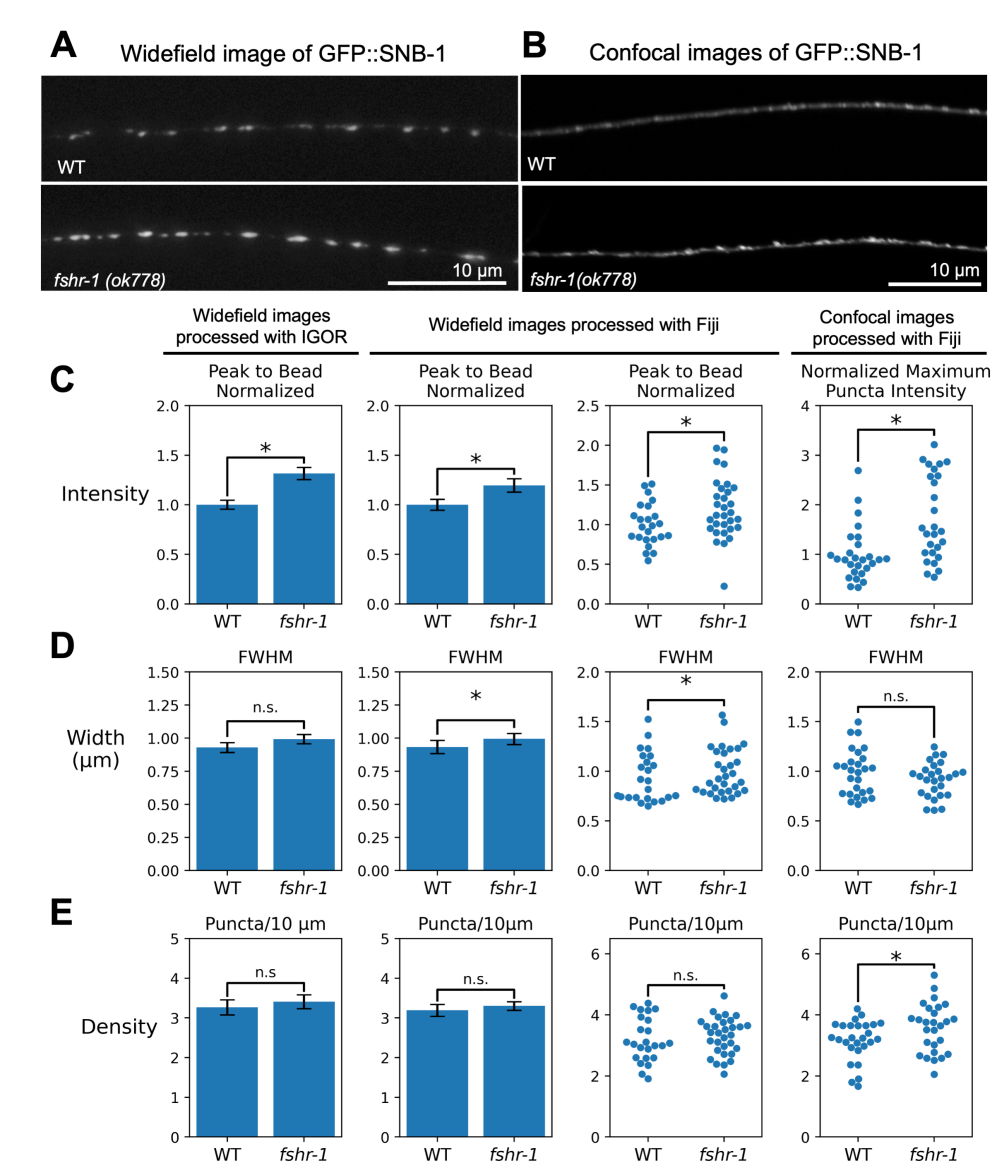
**A, B.** Representative images of WT and *fshr-1* mutant animals expressing GFP::SNB-1 imaged using widefield and confocal microscopy, respectively. **C** . Quantification of puncta intensity (peak-to-bead) per animal. Left, original quantification using IgorPro software; Center; analysis of the same data set using the Fiji macros (shown as bar graphs, with error bars representing SEM, left, and swarm plots, right); Right, quantification of images acquired using confocal microscopy by the Fiji macros displayed as swarm plots to show normalized intensity values for individual animals. Statistics for all plots are described in the text. **D, E** . Quantification of puncta width measured as Full-width Half-maximal (FWHM) fluorescence (D) and puncta per 10 𝜇m (E).

## Description

Understanding the molecular mechanisms controlling changes in neuronal signaling is critical to a complete understanding of nervous system function and, thus, essential for our ability to treat neurological conditions of all types. One way to assess the structure of synapses in *C. elegans* is by imaging fluorescently labeled synaptic proteins. A well-established example is green fluorescent protein-tagged SNB-1/synaptobrevin (GFP::SNB-1), a marker of synaptic vesicles (Nonet, et al., 1998; Sieburth, et al., 2005) . Quantitative imaging of GFP::SNB-1 has been used extensively as a measure of synaptic vesicle localization at neuromuscular junctions (NMJs) in the dorsal nerve cord of *C. elegans,* where it is expressed under various cholinergic or GABAergic motor neuron-specific promoters (Sieburth, et al., 2005; Vashlishan, et al., 2008; Dittman and Kaplan, 2006) . Images of the dorsal nerve cord of animals expressing one of the neuron-specific GFP::SNB-1 transgenes reveal the localization of GFP::SNB-1 proteins in discrete puncta that have been shown to mark synaptic sites given their colocalization with presynaptic active zone proteins and postsynaptic markers (McEwen et al 2006) . Changes in the density of GFP::SNB-1 thus are thought to correlate with changes in synapse number. Likewise, changes in GFP::SNB-1 puncta intensity seen in various endo- and exocytic mutants correlate with changes in synaptic vesicle number by electron microscopy (Sieburth, et al., 2005; Richmond, et al., 1999; Weimer et al., 2003) . Finally, increases in puncta intensity, indicative of increased synaptic vesicle accumulation and decreased vesicle release, often correlate with decreased signaling in electrophysiological studies (Kowalski, et al., 2014) , indicating the functional relevance of GFP::SNB-1 imaging.

Despite the utility of GFP::SNB-1 imaging to understanding synapse structure and function, to date, quantification of the above parameters and others, such as synapse size (full width half max, FWHM), has required the use of Igor custom-written software that could process and analyze linescan data taken from maximum intensity projections of dorsal nerve cords (Burbea, et al., 2002) . Here, we demonstrate how a new puncta analysis protocol for use in Fiji (Schindelin, et al., 2012) can be used to assess the same parameters using an open source, freely available software package (Hulsey-Vincent, et al. B, 2023) . We employed Fiji macros for linescan analysis of dorsal nerve cord GFP::SNB-1 puncta that provide outputs of synapse number and relative changes in GFP::SNB-1 puncta intensity and size. Results of this analysis align with previously published data, as well as with our own newly independently acquired data analyzed via the customizable Igor software.

To validate the Fiji puncta macros for GFP::SNB-1, we utilized the well-characterized strain *nuIs152* , which expresses GFP::SNB-1 under *Punc-129,* a promoter that drives expression in a subset of DA and DB cholinergic motor neurons (Sieburth, et al., 2005) . Along with imaging wild type transgenic animals, we also imaged GFP::SNB-1 in *fshr-1* ( *ok778* ) loss of function mutants. These are mutants of interest to our lab that have been shown to impact GFP::SNB-1 localization in both a prior published screen (Sieburth et al, 2005) and in our own hands. Specifically, *fshr-1(ok778)* mutants were previously reported to have increased GFP::SNB-1 puncta intensity, which correlates with resistance to paralysis induced by the acetylcholinesterase inhibitor, aldicarb, suggesting decreased vesicle release (Sieburth, et al., 2005) .

We imaged *nuIs152* and *nuIs152;fshr-1(ok778)* animals by both widefield ( *nuIs152,* N = 24; *nuIs152;fshr-1(ok778),* N = 31),and spinning disk confocal fluorescence microscopy ( *nuIs152,* N = 27; *nuIs152;fshr-1(ok778),* N = 27), Figure 1A and B . For the widefield data, both the customized Igor analysis software and our new Fiji macros measured a similar increase in GFP::SNB-1 puncta intensity in the *fshr-1* mutants compared to the wild type animals without any significant changes in puncta width or synapse number per micron (puncta density). We analyzed puncta intensity by normalizing to beads and found that the peak to bead increased in *fshr-1* mutants ( Figure 1C, T -test, IGOR: 32% increase, p=0.0022;Fiji: 20% increase, p=0.034). There was no significant change in puncta density (T-test, IGOR: p=0.81, Fiji: p=0.54). Puncta width was very slightly increased in the *fshr-1* mutants, but these results were only statistically significant when analyzed using the Fiji protocol (K-S test, Figure 1D, IGOR : p=0.84, Fiji: 6% increase, p=0.042). Importantly, we found good corroboration of synapse number ( Figure 1E, ~3 synapses/10 µm) in the *nuIs152* wild type strain with that published previously by our lab and others (Vashlishan, et al., 2008; Kowalski, et al., 2014; Kreyden, et al., 2020) .

To demonstrate the utility of the Fiji macros for analyzing data acquired across microscope and acquisition software types, we also compared results of *nuIs152* and *nuIs152;fshr-1* imaging done on a spinning disk confocal in our laboratory and found similar increases in GFP::SNB-1 puncta intensity in the *fshr-1* mutants, as well as similar synapse numbers to those published previously. For images acquired using confocal microscopy, the average maximum puncta intensity in *fshr-1* mutants was significantly higher than in wildtype (72% increase, K-S test, p=0.00048). Notably, the values per worm are very broadly distributed in the *fshr-1* mutant, which may reflect the reduced background in confocal images. Unlike peak intensity, we did not detect a change in puncta width between the wild type and *fshr-1* mutant animals (T-test, p=0.18).

Despite the similar results for mean puncta intensity and width between the widefield and confocal datasets analyzed with the Fiji macro, confocal microscopy uncovered a small, but significant increase in puncta density in animals harboring the *fshr-1* mutation (13%, K-S test, p=0.022). To address whether these differences represent dataset-specific variability, we performed the same analyses on two other independent datasets. The second set of analyses suggests that while there does appear to be variability between individual data sets, the overall patterns and trends are maintained (see Extended data). Specifically, in the secondary widefield datasets, there were modest but significant differences in puncta width (16% increase, IGOR only, T-test,p=0.013) and puncta density (11% decrease, Fiji only, T-test, p=0.026) for *fshr-1* mutants compared to wild type animals (Extended Data Figure 1 ). The secondary confocal dataset again showed a large and statistically significant ~70% increase in puncta intensity (p = T test, p=3.0e-07) in the *fshr-1* mutants with no significant differences in synapse width or density (Extended Data Figure 1 ). Thus, there are modest differences in density and width between the original and extended datasets, as well in between analyses run using Igor vs. Fiji. These differences suggest some variability likely based on image quality (widefield vs confocal) and analysis method. However, the most robust difference (i.e., puncta intensity) was reproducible across both imaging and analysis platforms. These findings suggest that while caution should be taken in over-interpreting small differences with marginal *p-* values on any platform, the Fiji plug-in is as reliable as Igor for detecting differences in synaptic protein abundance and distribution.

Together, these data demonstrate the utility of the Fiji puncta plugin for use with cross-platform GFP::SNB-1 imaging in the dorsal nerve cord motor neurons, augmenting its use in GLR-1::GFP quantification (Hulsey-Vincent, et al. A, B, 2023) . Improved image quality and lower background fluorescence achieved with the confocal microscope likely contribute to the marginally greater increases in *fshr-1* mutant peak intensities, as well as to slight variations in the other parameters in the confocal dataset. Additionally, it should be noted that across both widefield and confocal microscopes, we do observe some modest variability in the extent of differences between wild type and mutant animals when comparing different experimental datasets. Importantly, however, the parameters showing most significant increases or decreases and the direction of those changes (in this case, increased GFP::SNB-1 puncta intensity in the *fshr-1* mutants) remain consistent regardless of microscope type or analysis method. Thus, we expect that the open-source Fiji puncta plugin will be similarly useful for analyzing other pre- and postsynaptic markers at neuromuscular junctions, including active zone proteins and postsynaptic receptors.

## Methods

***Strains and Strain Maintenance***

All strains were grown at 20°C on plates containing nematode growth medium (NGM) agar spotted with OP50 *Escherichia coli* as a food source as previously described (Brenner et al. 1974). Strains used in this study include *nuIs152 (Punc-129::GFP::SNB-1),* JRK42 *nuIs152;fshr-1(ok778).* Young adult hermaphrodites were used for all experiments.

**Image processing**

Images (widefield and confocal, see below) were cropped and set to matching input levels using Adobe Photoshop (Kowalski, et al 2014) . Representative images were finalized in Microsoft PowerPoint by adjusting sharpness and contrast for clarity in figures. All adjustments were made uniformly for all figures.

***Quantitative Widefield Imaging***

Wild type and *fshr-1* mutant worms carrying the GFP::SNB-1 transgene in DA and DB motor neurons ( *nuIs152* ) were immobilized in 30mg/mL 2,3-butanedione monoxime (BDM) in M9 on a coverslip and mounted on slides containing 2% agarose pads. Quantitative imaging of GFP::SNB-1 fluorescent puncta in the dorsal nerve cord of worms oriented “dorsal up” (in which the dorsal nerve cord was facing the coverslip side closest to the objective) was performed using a Leica DMLB microscope (Leica Microsystems) with a 100x Plan Apochromat (1.4 NA) objective equipped with green and red fluorescence filters. Images were captured using an Exi Aqua cooled CCD camera (Qimaging) with Metamorph (v7.7) software (Molecular Devices) as described previously (Kowalski, et al., 2014; Kowalski, et al., 2020) . Maximum intensity projections of z-series stacks (0.2 μm steps, 1 μm total depth, 6 planes total) were generated from each stack. Exposure settings, gain, and binning were set to fill a 12-bit dynamic range without saturation (2x2 binning, 50 ms exposure for focusing; 1x1 binning, 150 ms exposure for imaging). Mercury arc lamp output was normalized by measuring the intensities of 0.5 μm FluoSphere beads (Invitrogen Life Technologies) at 1x1 binning, 2 ms exposure, for each imaging day. On any single day of imaging, at least three images of each strain were obtained in order to account for daily variation in the samples.

***Quantitative Confocal Imaging***

Wild type or *fshr-1* mutant worms carrying the GFP::SNB-1 transgene ( *nuIs152* ) were immobilized using 30 mg/mL BDM in M9 on a coverslip which was then mounted onto a glass slide containing a 2% agarose pad. Imaging was performed on a Nikon Yokogawa Spinning Disk Field Scanning Confocal Microscope equipped with Nikon Elements software. The worms were found and marked using a 10x EC Plan-Neofluar 10x/0.30 NA objective and then imaged using a 100x Plan-Apochromat (1.4 NA) oil objective. Only “dorsal up” worms, as described above, were imaged. Images were taken using the 488 nm laser microscope set to 26.9% power. A 100 ms exposure time and 1x1 binning were used for focusing, 300 ms exposure, and 1x1 binning for image acquisition. Images were taken over a total depth of 1µm, with a step size of 0.1µm for a total of 11 planes, which were compiled to make a single maximum-intensity projection. Approximately 20-30 maximum intensity projection images, one image per worm, were obtained for each strain. On a single day of imaging, at least three images of each strain were obtained in order to account for daily variation, as in with the widefield imaging.

***Metamorph/Igor-based Puncta Analysis*** ***(Widefield Imaging)***

Linescans of dorsal nerve cord puncta were generated using Metamorph (v7.1) software, and the linescan data were analyzed with Igor Pro (Wavemetrics) using custom-written software as previously described (Burbea et al 2002) . Puncta intensities were calculated by dividing the average maximal peak intensity by the average fluorescent bead intensity for the corresponding day. Puncta widths were determined by measuring the width of each punctum at half the maximum peak fluorescence (FWHM). Puncta densities were determined by quantifying the average number of puncta per 10 μm of the dorsal nerve cord. For all data, an average of the values for each worm in the data set ± s.e.m. is reported. Statistical significance of any differences between wild type and transgenic strain values was determined in Igor using a Kolmogorov–Smirnov Test or Student’s T test. Graphs of puncta intensities and width show data normalized to wild type values.

***Fiji-Based Data Analysis (Widefield and Confocal Imaging)***

Widefield puncta characterization with Fiji was performed as described in (Hulsey-Vincent, et al. A, 2023) with the following settings: minimum puncta size = 0.3, sigma = 0.75, radius = 1, method = Phansalkar. Confocal puncta characterization with Fiji was performed as described in (Hulsey-Vincent, et al. A, 2023) with the following settings: minimum puncta size = 0.3, sigma = 0.75, radius = 1, method = Phansalkar. Each variable measured was tested for normality using the Shapiro-Wilks test (α = 0.05). If the data were normally distributed, significant differences between strains were tested for using a Student’s T-test. If the data were not normally distributed, significant differences were tested for using a Kolmogorov-Smirnov Test.

## Reagents

**Table:** 

Strain name	Genotype	Available from the CGC
KP3814	*nuIs152 (Punc-129::GFP::SNB-1)*	Yes
JRK42	*nuIs152;fshr-1(ok778)*	No (Kowalski JR, et al. 2014)

## Extended Data

Description: nuIs152 and nuIs152;fshr-1(ok778) animals were imaged by both widefield (nuIs152, N = 29; nuIs152;fshr-1(ok778), N = 26), and spinning disk confocal fluorescence microscopy (nuIs152, N = 28; nuIs152;fshr-1(ok778), N = 36). One wildtype image was discarded from the Fiji dataset, since the script did not detect any puncta, likely due to the high background signal. For the widefield data, the customized Igor analysis software and the Fiji macros measured a similar increase in GFP::SNB-1 puncta intensity in the fshr-1 mutants compared to the wild type animals. We analyzed puncta intensity by normalizing to beads and found that the peak to bead increased in fshr-1 mutants (Figure 2A, K-S test, IGOR: 48% increase, p=0.00010; Fiji: 36% increase, p=0.00085). In these data, Igor detected a moderate but significant increase in puncta width in fshr-1 mutant animals compared to wild type, while Fiji did not (Figure 2B, T-test, IGOR: 16% increase, p=0.02, Fiji: p=0.21). Fiji detected a small decrease in puncta density for fshr-1 mutants, while IGOR did not (T-test,IGOR: p=0.89, Fiji: 11% decrease, p=0.026). Synapse number was still ~3/10 µm (Figure 2C). For images acquired using confocal microscopy, the average maximum punctual intensity in fshr-1 mutants was significantly higher than in wildtype (74% increase, T-test, p=3.0e-07). We did not detect a change in puncta width between the wild type and fshr-1 mutant animals (K-S test, p=0.32) or puncta density (T-test, p=0.096).. Resource Type: Image. DOI: 10.22002/h75vg-1ch76
